# How do metacognitive beliefs about memory differ between older adults with low and high dementia worry? A focus group study

**DOI:** 10.1136/bmjopen-2024-097002

**Published:** 2025-10-02

**Authors:** Astrid Emilie Lund, Alexandra Freiin von Stein zu Nord- und Ostheim, Hayley Ridley, Kamilla Bobyreva, Juliet L H Foster, Charlotte Russell

**Affiliations:** 1 Department of Psychology, King’s College London, London, UK

**Keywords:** Aging, Cognition, Dementia, QUALITATIVE RESEARCH, Old age psychiatry

## Abstract

**Abstract:**

**Objectives:**

This study aimed to examine how older adults form beliefs about their memory and how these beliefs are influenced by their level of concern about dementia. Inaccurate beliefs and excessive worrying, indicative of erroneous metacognition, are associated with negative health outcomes. This research can help identify mitigation for these harmful effects.

**Design:**

Qualitative focus groups; thematic analysis.

**Setting:**

Focus group discussion with healthy older adults hosted at a university in central London.

**Participants:**

35 healthy older individuals (women=29) without any psychiatric or neurological diagnoses, over the age of 65 years (mean 75.31, SD: 6.15). 13 participants were identified as having a high level of worry about dementia and 22 as having low worry. Groups were matched for cognitive performance on the Telephone Mini Addenbrooke’s cognitive assessment (Tele-MACE).

**Outcome measures:**

Participants were assigned to a focus group depending on their level of worry about dementia. During focus groups, a vignette prompted discussion around lifespan changes in memory and how this affected concerns around memory. This allowed investigation of the differences in beliefs about memory.

**Results:**

Thematic analysis revealed two key themes. First, older adults appear to base their definition of ‘normality’ of their own memory on comparisons. These comparisons were between themselves and others and between themselves now and their own past self. Despite similar strategies to define ‘normality’, those with high dementia worry had stricter definitions of what they determined as normal. The second theme described narratives around the ‘self’ and the ‘other’. There was a difference between those with high versus low worry; those with high worry had a strong focus on the ‘self’, while those with low dementia worry focused on ‘others’.

**Conclusion:**

Comparison is a common metacognitive strategy used in forming beliefs about memory. Targeting the use of comparison is potentially valuable in interventions aiming to alleviate older adults’ memory concerns. Addressing self-focused thinking, for example, with cognitive behavioural therapy, could improve harmful levels of high worry.

**STRENGTHS AND LIMITATIONS OF THIS STUDY**

- Participants were distinguished by their level of worry about dementia but were matched on gender, age, ethnicity and cognitive abilities.
- Vignettes were used to facilitate open discussions without necessitating participants sharing personal information.
- People who specifically avoid discussions around dementia and memory due to fear were unlikely to participate in this study.
- Measures of generalised anxiety and depression were not used.

## Introduction

 There has been an increased focus on dementia and its impacts across society, encompassing politicians, the media and general public.^1 2^ This focus has been accompanied by raised worry across the population about dementia, and dementia is now the second most feared disease after cancer.^3 4^ Some individuals are more burdened by these worries than others, which can be associated with negative life outcomes, such as poor mental health^5^,^7^ and, crucially, future cognitive decline.^8 9^ The fear surrounding dementia in healthy older adults is likely fuelled by uncertainty, as many older adults struggle to determine whether their memory slips are signs of early dementia or simply a part of ageing. Here, we aimed to investigate how people evaluate their memory and how this might vary according to the amount of worry about dementia, as this may help identify points of intervention for negative self-beliefs.

### The importance of metacognitive beliefs in older age

Subjective cognitive decline is a clinical diagnosis describing individuals who think they are experiencing cognitive decline but do not show any impaired cognitive abilities when assessed.^10 11^ It has been found that 25–50% of the older population experience such subjective cognitive decline.^12 13^ The cognitive skill we use to make such subjective judgements of our cognitive abilities is called metacognition.^14 15^ In older age, metacognitive beliefs of memory seem to be particularly important, as memory impairment is the key characteristic of dementia.^2^ The high prevalence of subjective cognitive decline, therefore, suggests that metacognitive inaccuracies are common in older age and clinically relevant. For instance, it has been estimated that approximately 20–30% of those who seek assessment at memory clinics have subjective cognitive decline in the absence of objective cognitive impairments.^16 17^ This highlights the burden that these inaccurate metacognitive beliefs can have on the healthcare system and therefore the importance of understanding why such beliefs occur. Crucially, inaccurate metacognitive beliefs also burden and provide a health risk to the individual, as they have been associated with poor well-being, mental health difficulties and higher mortality rates.^5^,^717^ Research even suggests that negative metacognitive beliefs of cognitive decline are associated with future cognitive decline.^1021^,^25^ It is therefore important to understand what causes these negative and inaccurate metacognitive beliefs surrounding memory abilities.

### Reasons for inaccurate metacognitive beliefs in older age

While up to half of older adults have worries about their memory abilities and show signs of subjective cognitive decline,^12 13^ only around 5% will go on to develop Alzheimer’s disease.^26^ Therefore, for many people, these worries of dementia might be unfounded. One such reason for the high level of worry may be the difficulty in distinguishing typical age-related decline from pathological memory changes. For example, semantic memory, our knowledge including memory for facts, appears to be preserved and may even be enhanced in older age,^27^ while episodic memory, our memory of events, does tend to decline.^28^ These differential patterns of decline in episodic and semantic memory may explain some of the difficulties older adults face when trying to make metacognitive judgements of their cognitive state. In addition, episodic memory difficulties are the first sign of the most common form of dementia—Alzheimer’s disease.^2 29^ Knowledge of dementia varies between individuals and cultures, and greater awareness is encouraged.^30 31^ However, research suggests that many older adults know that incidents of forgetting what has happened or where one is may be signs of dementia.^32 33^ Both of these are examples of episodic memory slips. Thus, it seems plausible that older adults may be particularly wary about changes of this type. Furthermore, if a person notices such changes, it is likely that they will change their metacognitive beliefs about their ability to remember and may start to worry about dementia.

Another reason for inaccurate metacognitive beliefs may be the reliance on ineffective metacognitive strategies. Metacognitive strategies are the processes used to develop a specific belief. When it comes to metacognitive beliefs about one’s general traits, research suggests that one such metacognitive strategy is social comparison. This is the comparison we make between peers’ and our own ability; the comparisons have been shown to influence a person’s self-belief.^34 35^ If these comparisons are made in a maladaptive manner, such as overestimating the discrepancy between oneself and one’s peers, it may lead to metacognitive inaccuracies. In the current study, we aimed to understand some of these underlying reasons for metacognitive inaccuracies in older age, centring the experiences of older individuals through focus group discussions. In particular, we were interested in investigating how older adults may form their metacognitive beliefs based on different types of memory information, such as information from experiences of episodic and semantic memory slips.

### The relationship between metacognitive beliefs and dementia worry

Evidence suggests a high association between fear of dementia and inaccurate metacognitive beliefs in older age.^36^,^41^ The exact causal relationship between negative metacognitive beliefs and fear of dementia is not understood. However, it has been found that worry about dementia is associated with interpreting memory slips as more negative, leading to the tendency to seek out signs of cognitive decline in one’s own behaviour.^42 43^ This suggests that dementia worry is one possible factor underlying the development of inaccurate metacognitive beliefs in older age. A theoretical framework of dementia worry was set out by Kessler and Bowen,^44^ who suggested that the level of dementia worry is caused by a negative perception of (1) the risk of getting dementia, (2) the seriousness of the condition and (3) the access and effectiveness of coping mechanisms (eg, see the study by Maxfield *et al*^45^). Here, we will investigate people’s level of dementia worry in order to probe how it interacts with metacognitive strategies and beliefs about memory in older age. Understanding what influences metacognitive beliefs, and how this interacts with fear of dementia worry, may help us identify points of intervention to decrease the negative subjective beliefs and therefore minimise the associated burden to the individual and healthcare system.

Here, we conducted focus groups with adults aged 65 years and above. In order to understand how different levels of worry might lead to differing perceptions of memory, separate focus groups were run with individuals who showed respectively low and high worry of dementia. All groups were structured around a fictional vignette. The discussions were analysed with thematic analysis,^46 47^ as this is a widely used analysis framework in qualitative work and allows for the flexibility and depth of analysis we aimed to achieve.

## Methods

### Participants

35 healthy participants (women=29) aged 65–85 years of age (mean=75.31, SD=6.15), without any current psychiatric or neurological diagnosis were recruited. Participants were recruited through internal advertisement at King’s College London and external advertisement at University of Third Age. Participants provided informed consent. Based on their scores on the Modified Dementia Worry Scale,^48^ participants were divided into high dementia worry and low dementia worry. 22 participants were identified as having low worry (mean=18.14, SD=4.54) and were divided into another four focus groups (four to seven participants in each group). 13 participants were identified as having high worry as they had scores over 30 (out of 60) (mean=36.08, SD=4.94) and were divided into three focus groups (three to four participants in each group). Participants were added to a group based on dementia worry level and availability for partaking in focus group discussions. The groups were matched on gender, age, ethnicity and cognitive ability as measured by the Telephone Mini Addenbrooke’s cognitive assessment (Tele-MACE) (see table 2). Previous studies suggest that three to four focus groups are adequate for thematic saturation; therefore, the study is sufficiently powered.^49 50^ Ethical approval was received from King’s College London Ethics Committee (ref: HR/DP-21/22–26212).

### Patient and public involvement

Participants were not involved in development of the study design. If participants indicated on the consent form they wished to receive the final manuscript, they will be provided with this via email.

### Materials

#### Telephone Mini Addenbrooke’s cognitive assessment

The Tele-MACE^51^ is a short telephone-adapted version of the standardised MACE, designed to assess cognitive ability. Participants completed four tasks testing memory, verbal fluency and attention. The maximum score was 25, with a threshold of 19 indicating mild cognitive impairment.

#### Modified Dementia Worry Questionnaire

The Modified Dementia Worry questionnaire (mDMQ)^48^ is a 12-item questionnaire assessing the level of worry about dementia. Participants rate statements such as, “When I forget a word that I want to say, my thoughts immediately turn to Alzheimer’s disease and related dementias” on a scale from 1 ‘not at all typical of me’ to 5 ‘very typical for me’. A score over 30 of 60 points was defined as high worry.

#### Behavioural questions

Two questions were used to assess whether participants had sought advice due to memory worry. First, participants were asked to answer, ‘yes’ or ‘no’ to the question, ‘Have you contacted your general practitioner or another medical professional because you were worried about your ability to remember?’. Second, they were asked to rate the question, ‘How often have you talked to friends, family members, or another close relation because you were worried about your memory?’, on a scale from 1 ‘never’ to 5 ‘very often’.

#### Metacognitive memory question

One question directly assessed participants’ metacognitive judgements of their memory ability. Participants were asked to rate on a scale from 1 ‘not very good’ to 5 ‘extremely good’: ‘Please indicate how good you think you are at remembering things’.

#### Vignette

A vignette was developed to facilitate the focus group discussions (see table 1). This was chosen as it allows focus on the fictional characters rather than on oneself.^52^ In addition, a vignette removes the focus from the researchers, minimising demand characteristics and potential outsider issues caused by age differences.^52^

**Table 1:** Procedure of the focus group discussions in chronological order

Icebreaker	Which animal would you use to describe your morning routine?
Vignette	Mary lives in London, and she has two grandchildren whom she loves dearly. She visits them each Saturday, and they usually go to the park to look at the ducks. Mary has to take two tubes to get to their place, but she knows the route and is never late. She always makes sure to bring a few sweets that she gives to the grandchildren when they are in the park, so their mother does not notice. This Saturday, her tube journey goes smoothly, and her grandchildren are putting on their jackets when she arrives. On their way to the park, they meet an old friend of Mary’s, Paul, who is walking his dog. Mary and Paul talk for a bit, while the grandchildren pet the dog. Afterwards, they find the pond, which is crowded with ducks. The grandchildren ask Mary what the baby ducks are called, and she says they are called ‘ducklings’, and they count that there are seven of them. After a while, Mary puts her hands in her pockets to get the sweets for her grandchildren but realises that she has forgotten them. The grandchildren laugh and say it is okay. To make up for the missing sweets, Mary suggests that they buy ice cream instead, before they go home.
Vignette questions	1.1. How do you think Mary feels about forgetting the sweets? 1.2. Do you think forgetting the sweets will affect Mary in any way next time she takes her grandchildren to the park? 1.3. Do you think forgetting the sweets affects how Mary thinks about her ability to remember? 1.4. Do you think Mary would feel differently had she forgotten the name of her old friend Paul? 1.5. Do you think Mary would have felt differently had she forgotten her tube route? 1.6. Do you think if Mary had forgotten that baby ducks are called ducklings, she would have been more or less concerned? 1.7. How old do you think Mary is? How does this influence your answers? 1.8. Do you think you would have answered differently if Mary had forgotten something in a work context instead of near her grandchildren? If, for instance, she had forgotten the keys to the charity shop where she volunteers. 1.9. If Mary starts worrying about her memory, do you think there is a way for her to regain her confidence?
General questions	2.1. Some individuals think they are bad at remembering even though they are not. Why do you think that is? 2.2. Based on our discussions today, can you list the two most important types of events that you think would cause someone to doubt their own memory ability? 2.3. Based on our discussions today, which aspect of a person’s life do you think is most affected by doubting their own memory?
Final question	3.1. What do you think was the most important or interesting point that was brought up today?

The vignette described the story of a fictional woman named Mary, who forgets things across different scenarios (see table 1 and online supplemental material 1 detailed protocol). These memory slips were developed to cover different aspects of memory, including semantic memory (eg, the word for baby ducks), episodic memory (eg, bringing keys and sweets), spatial episodic memory (tube route) and personal semantics (name of a friend^53^), as well as incorporating different contexts in which the events occurred (professional and familial). Many of the predetermined questions concerned the vignette scenario. Additionally, a few general questions were developed to gain insight into the participants’ perspectives on memory loss, and a final question was used to wrap up discussion (see table 1). Based on the progress of the conversation, some questions may not have been asked in all focus groups.

### Procedure

**Figure 1:**
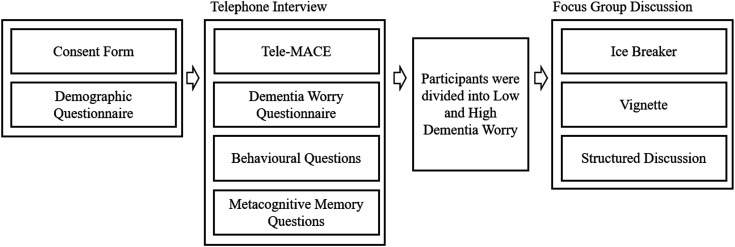
Procedure. Consent and demographics were done either independently by the participant online, or over the telephone. After division into groups (ie, low and high dementia worry) focus group discussions were held. Tele-MACE, Telephone Mini Addenbrooke’s cognitive assessment.

As seen in figure 1, participants filled in the consent form and provided demographic information regarding age, gender and ethnicity, which allowed screening for exclusion criteria (ie, no current neurological or psychiatric diagnosis, except subjective cognitive decline). These forms were either completed independently online or with a researcher on the telephone. All participants then completed a 15 min telephone interview, either immediately after having given consent or on a different day. During the telephone interview, the Tele-MACE^51^ and mDMQ Scale^48^ were administered. These were followed by the behavioural and metacognitive memory question.

Following the interview, the participants were assigned to focus groups based on their dementia worry scores. Participants were split between low and high worry to facilitate safe spaces for discussion with individuals with similar perception and worries regarding memory. In addition, it allowed us to investigate how the level of dementia worry influenced participants’ reports about their metacognitive beliefs and how they develop these beliefs. The focus groups lasted 1 hour long and were audio recorded. Focus group discussions started with an icebreaker (table 1). Following this, the researcher read the vignette aloud, which the participants had also been given in written form. The discussion was semistructured and guided by a researcher, who asked the questions provided in table 1 and provided prompts like, ‘Please will you elaborate on that point?’ or ‘Do the rest of you agree with this point?’, to guide and promote discussion among the participants.

## Qualitative methodology

An inductive form of thematic analysis, where the data guide the analysis,^46 47^ was conducted. This is a widely used method, which is a flexible yet comprehensive analysis framework. According to thematic analysis, five steps were followed: (1) familiarisation: the authors AEL, AFSNO, HR and KB read and went through transcription and audio recordings of the focus group discussions several times aided by the software otter.ai. (2) Coding: the transcripts were coded in the software NVivo V.14 by AEL. Codes are labels describing specific parts of the data, such as ‘reason for worry’. These codes are then compared and combined to create themes. Codes were created separately for each group (low worry and high worry). (3) Generating themes: initial subthemes were created first for each group (low and high dementia) and then overarching themes were created covering both groups. (4) Reviewing themes: the themes and subthemes were reviewed several times, based on discussions between all authors, alongside the re-reading of codes and texts. (5) Defining themes: the final themes and subthemes were defined to describe the data.

A detailed coding diary was kept by the first author, including reflections of construction of themes and sub-themes as well as reflexivity, positionality and potential biases.^54^ A comprehensive audit trail was kept through audio recording, documents and NVivo coding. The development of themes and subthemes was regularly discussed between AEL, JF and CR, to facilitate critical feedback and reflection. In addition, these were shown to AFSNO, HR and KB who provided feedback. Finally, the study was retrospectively reviewed against the Consolidated Criteria for Reporting Qualitative Research checklist to ensure comprehensive reporting and reflection of each point;^55^ see online supplemental material 2. The sample was appropriately powered to achieve thematic saturation.^49 50^

### Quantitative methodology

Quantitative differences between the two groups for the questionnaires and cognitive assessment were analysed. It is important to note that the aim of the current study was to examine qualitative differences between these groups, the quantitative results should be considered in this light, as the sample is slightly skewed (more people with low worry) and underpowered for quantitative analyses. Assuming a medium effect size of d=0.05, the power to detect this difference in a two-tailed t-test was β=0.28, well below the standard of β=0.80. Conservative non-parametric tests (Wilcoxon Signed Rank test and χ^2^ tests) were employed to minimise these issues. The tests were used to ensure that the groups were appropriately matched and investigate differences between those with low and high dementia worry. Given the lack of power, these results should be interpreted cautiously.

## Results

### Quantitative group differences

Based on mDMQ scores, participants were divided into three groups of individuals with high worry (n=13) and four groups of individuals with low worry (n=22). As groups were defined by scores on the mDMQ,^48^ a significant difference was found between the groups on this scale (W=286; p<0.001). In addition, participants in the high worry group were significantly more likely to have discussed their dementia worry with relatives or a clinician (table 2). No difference was found between the two groups in their daily confidence about memory (see table 2). Importantly, as shown in table 2, there was no statistical difference in Tele-MACE scores between the groups, suggesting that any increased worry is not a result of memory decline in any of the group participants. One participant in the low worry group scored below the threshold for mild cognitive decline (MCI) (<19), and one participant in the high worry group reported serious cognitive decline during the focus group. However, as neither of these constitutes an MCI diagnosis and statistical results remained stable with and without these participants (see online supplemental material 3), they were retained in the study.

**Table 2:** Mean and SD of questionnaires and cognitive assessment; dependent 2-group Wilcoxon signed rank test for numeric variables; χ^2^ tests for categorical variables

	Low (n=22)	High (n=13)	Total (n=35)	Significance
Gender: women (men)	14 (8)	12 (1)	26 (9)	0.140
Age (SD)	76.29 (6.50)	73.64 (5.33)	75.31 (6.15)	0.371
Ethnicity: white (other)*	18 (3)	11 (2)	29 (5)	1
Tele-MACE (SD)	23.27 (2.05)	23.92 (1.11)	23.52 (1.77)	0.54
Dementia worry (SD)	18.14 (4.54)	36.08 (4.94)	24.8 (9.93)	<0.001†
Memory confidence (SD)	3.18 (0.73)	3 (1.08)	3.11 (0.87)	0.617
Subjective cognitive decline diagnosis:	No: 22 (yes: 0, I do not know: 0)	No: 12 (yes: 0, I do not know: 1)	No: 34 (yes: 0, I do not know: 1)	0.787
Discussion with relatives (SD)	1.95 (0.84)	2.77 (1.09)	2.26 (1.01)	0.022†
General practitioner: yes (no)	0 (22)	8 (5)	8 (27)	0.008†

* Ethnicity, Other: Indian (n=2), Asian (n=1), Jewish (n=2). Unknown (n=1).

† Significant at p<0.05.

Tele-MACE, Telephone Mini Addenbrooke’s cognitive assessment.

### Qualitative group differences and similarities

Through the iterative process of thematic analysis, themes were created across groups, with subthemes defined as either shared or distinct between low and high worry groups (see table 3). Below, participants are given identifying codes to represent both their group and gender: LW for low worry; HW for high worry; F for female; M for male, along with a unique number.

**Table 3:** Theme and subthemes defined based on the group discussions

Themes	Group	Subthemes
1. The question of normality	Shared	1.1 Strategies for defining normality 1.2 Characteristics and threshold of normality 1.3 Preservation of normality
	Low worry	1.4.a The normalisation of forgetting
	High worry	1.4.b The normalisation of fear
2. The importance of the self or others	Low worry	2.1.a An issue relevant to others 2.2.a Consequences of dementia on others 2.3.a Coping with potential dementia
	High worry	2.1.b An issue relevant to oneself 2.2.b Consequences of dementia on the self 2.3.b Fear of potential dementia

The two groups (low and high worry) share themes, while subthemes were either shared or split between the two groups.

#### Theme 1: the question of normality

The theme of normality was raised by all groups but discussed in different ways. This theme refers to how an individual’s perception of normality determines what is deemed as worrisome. This theme features three cross-group subthemes, some of which were shared while others were split between the groups (See table 3).

##### 1.1 Strategies for defining normality (shared)

Both groups discussed the types of metacognitive strategies they used to build their metacognitive beliefs about their own (and others’) memory ability. In particular, participants reported relying on comparison. For example, comparing themselves to others:

LWF2: I think another element that Mary could be thinking of is, is, how her forgetfulness compares with other people’s forgetfulness. If she finds other people not remembering duckling or the word, then she wouldn’t feel that she was anything other than normal.

The quotation above shows how such comparison can give comfort, if they recognise the behaviour as normal. However, if recognised as different from others, this could also lead to worry: ‘But I’m concerned because of the way I am. And comparing with my wife who is 5 days older than me. Her memory is 100%.’ (HWM1). Participants, however, also recognised that due to individual differences, comparing oneself to others may be misleading at times. They therefore also compared themselves to their past selves:

HWF2: Yes. I’ve always been terrible with names. […] I think it would depend again, what her normal memory for names was, like so much of it is comparative, that it’s not actually what happens once, it’s how often it happens and how it relates to how you were in the past.

Participants also discussed the limitation in building one’s metacognitive beliefs on one’s own observations. Many participants therefore highlighted the importance of input from other people to inform their beliefs: “I find [that] on my own, I get away with murder. […] Nobody notices, so I don’t notice; So, in a way, I’m a bit less aware of sinking into a memory loss” (LWF3). Overall, both groups reported primarily relying on comparisons to other people or their past selves. However, the groups differed in whether this comparison comforted them or added to their worry.

##### 1.2 Characteristics and threshold of normality (shared)

Both groups discussed the metacognitive beliefs or criteria they had for ‘normal’ memory. First, events which can be explained by external factors were not deemed as worrisome, such as “distraction” (LWM1) and mood:

HWF1: I think it also depends on your kind of mood of the day […]. If you’ve got things on your mind […]. If it [edit: the brain] gets overloaded. And I think that’s quite interesting. Because I know that happens to me if I have too much going on, other things can go out the window.

Additionally, transient forgetting was seen as normal: “I don’t get stressed. I know it will come back because it’s in your head. It’s not something you’ve forgotten forever.” (HWF5). Generally, most of the scenarios presented were only deemed worrisome if they started to accumulate: “…if it keeps recurring, the next time and so on, but that’s when the worry starts to kick in, I think” (LWM2). We did not see a strong difference in the interpretation of memory slips depending on whether these were episodic or semantic in general. However, the specific exception to this was the scenario of forgetting the tube route, which relies on episodic memory. All participants viewed this as immediately worrisome:

LWF2: That’s scary. If you find yourself somewhere that, I know I’ve got to go somewhere else. But where do I go? Yeah, that would be, that would be worrying. Frightening. I think. If you’re losing your bearings, what would it feel like?

When participants started to share their own stories, it became clear that participants with high worry had a higher threshold for normality, as they explicitly voiced their worries: “You know, is that normal? I’m not so sure at that stage.” (HWF8) and seemed to be aware that they might worry more than other people: “I think if Mary is somebody other than myself, I would feel the same [edit: not worry]. But if Mary was myself, I wouldn’t feel the same—I would worry” (HWF3). Thus, while participants mostly show similar interpretations of what ‘normal memory’ is in the main discussion, when participants started to share their personal stories, it became clear that the two groups differed in their definitions of normal/non-worrisome memory changes.

##### 1.3 Preservation of normality (shared)

Both those with high and low worry discussed the importance of prevention of cognitive decline to maintain normality. This was, for instance, by learning new things and solving cognitive tasks:

LWF8: It’s really important to learn languages and do mental things like Sudoku and cryptic crosswords and things, as I said, that it really does help people that are showing some symptoms, but I think it must, it must help your brain. Keep it alive.

Both groups therefore tried to prevent dementia to the extent that they could.

##### 1.4.a The normalisation of forgetting (low worry)

Participants in the low worry groups engaged in a process of normalising forgetting. In these groups, personal stories of forgetting were met by other participants with comments such as: “Oh, I do that all the time, and I don’t worry about it.” (LWF3) or “But we all forget things from time to time.” (LWF4). They therefore normalised forgetting, which was also seen in their way of normalising the use of coping strategies:

LWM3: But I mean what Mary could do is what everybody does. Is put a post-it note on the front door saying ‘put the sweets in your basket’. But that happens to me and I get the impression it happens to almost everybody, so should you worry about it?

Additionally, although they discussed heavy topics and expressed some degree of worry, they kept a light tone and even shared jokes: LWM3: “But isn’t there, that, the old joke about there are some benefits to getting old and having a bad memory, that you can watch a film for the second or third time”. These jokes are also a way of communicating that this is not an immediate worry to them. Interestingly, this normalisation of forgetting may be interpreted as avoiding the problem, these individuals also viewed denial of memory decline as very worrying: “Do you not agree? That is so worrying when people are in denial” (LWF7). Presumably, this was not viewed as contradictory, as they were less likely to consider themselves as having something to worry about (see the Consequences of dDementia on others (low w Worry) section).

##### 1.4.b The normalisation of fear (high worry)

Participants with high worry appeared to engage in a process of normalising fear. In these groups, when individuals shared personal stories about forgetting, such as getting lost in a park, these were often met with comments such as: “You are telling yourself: ‘is this normal?’ or ‘is that – it’s okay?’” (HWF6) or “That is disturbing […] but you forgive yourself” (HWF5). Thus, the participants’ reactions indicate that these situations are abnormal and should be a reason to worry. As seen in the quotations, they did however also try to positively influence each other by encouraging forgiveness. Although this also assumes something is wrong for which one needs to be forgiven.

### Theme 2: the importance of the self and others

The theme of the importance of the Self and the Others captures how the two groups discussed similar topics but leaned differently on this dimension, where those with high worry tended to highlight the importance of the self. Each subtheme had an opposite yet corresponding meaning in each worry group.

#### 2.1.a An issue relevant to others (low worry)

Participants with low worry seemed to view dementia as an issue that was not immediately relevant to themselves, but rather an issue concerning others. As seen in the subtheme Preservation of normality, they tended to maintain the sentiment not to worry about dementia and keep a light tone. Participants also shared fewer personal stories; instead, they focused on the vignette. The vignette did not include any explicit mentioning of dementia, and it took a while before the word ‘dementia’ was mentioned by participants (average LW group 26 min.; HW group: 20 min 35 s) and it was mentioned fewer times than by those with high worry (average 12.5 times per group; HW: 15.3 times). They were also likely to bring up the issue from the perspective of the family and close friends, rather than that of the person with dementia:

LWM5: Well, then you, you know, if you’re a close family, you work around it. Now that means come, come and be with the children here, rather than go out with them and things like that.

Participants therefore tended to view dementia as something *others* develop rather than themselves, therefore creating a distance between themselves and the disorder.

#### 2.1.b An issue relevant to oneself (high worry)

Those with high worry viewed dementia as an issue immediately relevant to themselves. For instance, they related stories of dementia back to themselves: “…because of my father [edit: who had dementia], I’m […] probably, I’m overcompensating and trying not to get dementia” (HWF1). Thus, clearly contemplating the possibility of them developing dementia themselves in the future. As proposed by one participant, the high worry may partly be caused by exposure to the disorder:

HWF8: I sometimes wonder if the reason why I’m worried is (a) because I’ve seen friends with dementia; and (b) because you just read more about it and we’re generally better informed.

Participants quickly removed their focus from the vignette to these personal stories, and the discussions therefore often surrounded dementia, although this was never mentioned by the researchers. These discussions were often more emotional and fearful, as seen in quotations like: “I’m afraid of it, I’m afraid.” (HWF3) or “I think the saddest thing is this. Sometimes there’s not a lot to look forward to.” (HWF7). Thus, these participants viewed dementia as a serious and relevant issue.

#### 2.2.a Consequences of dementia on others (low worry)

Those with low worry tended to focus on the consequences dementia can have on *others,* such as friends and relatives. A primary worry was therefore to be a burden: “It worries me being a burden” (LWF6). This also led to a distinction between ‘good’ and ‘bad’ dementia depending on the degree of harm the disorder would have to other people:

LWF6: And I think that’s the fear I have, is, going into dementia gently and being the dear old lady who can’t remember anything is fine. I don’t mind that. But I fear going into the, the other side where it’s inappropriate. And you disgust people.

As seen in the quotation, dementia was feared less if the harm to other people could be minimised. The reason why they focus on the consequences of dementia on friends and relatives may therefore be that this is whom they identify with (see the An issue relevant to others sectionIssue Relevant to Others ), rather than the person with dementia.

#### 2.2.b Consequences of dementia on the self (high worry)

Participants with high worry focused on the consequences dementia would have on the individual with dementia, that is, *the self*. Although those with high worry also expressed a fear of being a burden and the prospect of losing close relationships: “I do worry about getting it because I don’t want to be the one that is angry and […] change my relationship with my children and everybody” (HWF4), this was not emphasised as much, as by those with low worry. Instead, the focus was on the symptoms of the disorder itself and the risk of them getting it: “Her mum had dementia. Is she fighting? Is she aware? I don’t know. […] Sorry, it is just difficult to watch. And then you feel, am I going down that route too?” (HWF6). Those with high worry therefore did not draw a distinction between good and bad dementia, but instead were worried about dementia regardless of its specific symptoms.

#### 2.3.a Coping with potential dementia (low worry)

Participants with low worry mainly feared the potential harm on others caused by dementia. Participants even reported a wish for facilitated suicide, and thereby a sense of ‘sacrifice’ of their own self for the sake of others:

LWM1: …I certainly don’t want to be a burden to anybody. And I want to be able, I rather object to the fact that one can’t just say I want to end my life – facilitated, please. I think you should have that option.

Lesser coping strategies were also discussed as a useful and positive way to control and minimise that harm:

LWF7: There was an amazing woman with Alzheimer’s on television. She knows she’s got it […]. So, she knows exactly what to do if she forgets. […] all the cues, all the little notelets, or everything, post-its […] And she’s so content with herself, but she has Alzheimer’s quite badly. […] but how many people do that? Not a lot.

This ability to cope would minimise the prospect of being a burden to others and therefore provide a sense of hope and control for managing their fears associated with potential dementia.

#### 2.3.b Fear of potential dementia (high worry)

Participants with high worry appeared to focus on the consequences dementia would have to themselves, for which they perceived little or no relevant resources to cope. Instead of a fear of being a burden, these participants feared dementia itself (see the Consequences of dementia on the s Dementia on the Self section). As their worry is tied to dementia itself, and there is currently no way to fully prevent or treat dementia, these individuals understandably sense they have no means to manage the object of their fears. Coping mechanisms were therefore not a prominent point of discussion. Instead, the prospect of dementia and their potential future was a source of fear and uncertainty: “I’m afraid of it, I’m afraid.” (HWF3) and “When I get to 87, I don’t know where I’ll be. And that’s a little bit… That’s, that’s the worry” (HWF4). The focus on the self and therefore the perceived lack of relevant coping strategies appears to lead to decreased sense of control and increased sense of fear.

## Discussion

In this study, we aimed to qualitatively investigate how older individuals form their metacognitive beliefs about their memory ability. Employing thematic analysis,^46 47^ we defined two themes across both groups of low and high dementia worry. The first theme being “the question of normality” which incorporated analysis of older individuals’ metacognitive beliefs and strategies, and how this differs between individuals with low and high dementia worry. Strikingly, we found that despite the aim of the study being ‘thinking about memory’, the focus group discussions quickly evolved to ‘thinking about dementia’, leading to the definition of the second theme, ‘the importance of the self’. Here we discuss these findings in relation to previous literature on dementia and subjective beliefs about memory, as well as highlighting how these findings shed light on relevant targets for interventions regarding memory and dementia worries in older adults.

### Metacognitive beliefs and metacognitive strategies

Previous research has suggested that there is an association between inaccurate and negative metacognitive beliefs and dementia worry in older age,^36^,^41^ possibly leading to a vicious cycle of increasing worry and inaccurate metacognitive beliefs. However, this interaction between dementia and metacognitive beliefs has not been investigated in detail. In the current study, we found evidence that individuals with high dementia worry were more likely to exhibit negative beliefs about memory slips. On the other hand, those with low dementia worry tended to normalise situations of forgetting. Interestingly, the 1-item questionnaire on subjective beliefs about memory ability found no statistically significant difference between the low and high dementia worry participants. This question asked participants to rate their ability to remember. The fact that there is evidence of lower confidence around memory abilities among those with high worry in the qualitative analysis but not the quantitative analysis may result from a number of reasons. As previously mentioned, the sample was not powered for quantitative analyses but rather qualitative analysis. Issues around power for quantitative analysis are also exacerbated by the use of a 1-item questionnaire as there are a small number of data points. Conversely, the qualitative data are richer, allowing us to explore the nuanced differences between the two groups. It is worth noting that this limitation is common in this field of research; many studies investigating insight into memory abilities in older adults rely on 1-item questionnaires similar to the one used in this study.^21 56 57^ The current study highlights the need for richer research to assess subjective memory beliefs.

In the current study, we were particularly interested in how different types of memory may influence metacognitive beliefs. The only difference we found between the scenarios was, however, the scenario in which Mary got lost. All participants found this scenario to be frightening and reported that it would lead to a change in their metacognitive beliefs about their memory abilities. This is in line with the intimate relationship between spatial processing and the episodic memory system,^58^ of which the disruption is a core symptom of dementia.^59^ Other events of forgetting discrete semantic or episodic details were less associated with severe cognitive decline, and participants rightfully only reported this to be worrisome if such incidents started to accumulate. However, when the participants started to share their own personal stories of such incidents, those with high dementia worry often deemed these worrisome as well. In line with previous research,^42 43^ this suggests that individuals with high dementia worry may interpret memory slips more negatively.

Turning to metacognitive strategies, all participants reported relying on comparisons to develop their metacognitive beliefs about their own cognitive states. They mentioned both social comparison, that is comparison to their peers, which is in line with social comparison theories,^34 35^ but also mentioned comparisons to their younger selves as another metacognitive strategy. These comparisons were used to define their perception of ‘normal ageing’ and therefore the threshold for their worries. This suggests that those with high dementia worry may engage in maladaptive comparison strategies, leading to unhealthy perceptions of ‘normal ageing’, which in turn may lead to inaccurate metacognitive beliefs and dementia worry. We encourage future research to further investigate the causal relationships between comparison strategies, metacognitive beliefs and dementia worry, as this will be crucial for developing effective interventions.

### A self-other dimension as an important mechanism of dementia worry

**Figure 2:**
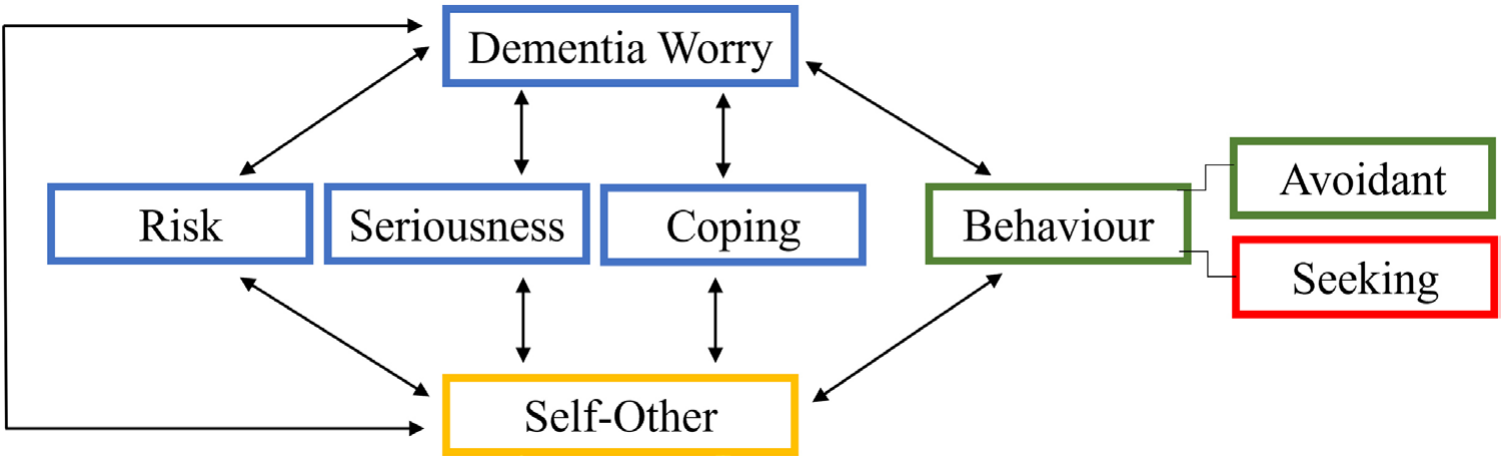
Blue: Original Model by Kessler *et al,*^44^ green: behavioural component by Werner *et al*,^7^ red: behavioural inspired by Edmonds *et al,*^97^ yellow: self-other dimension proposed by the authors of the current study.

In addition to findings related to differences in metacognitive beliefs dependent on dementia worry, our study also provided insight into the factors associated with dementia worry. Due to the negative outcomes associated with dementia worry, we argue that understanding the mechanisms of dementia worry is essential. Previous research has tried to explain some of the mechanisms associated with dementia worry. Most pertinently, the dementia worry model proposed by Kessler *et al*.^44^ They proposed that dementia worry depends on three dimensions: (1) the perception of risk of developing dementia, (2) the perception of seriousness of dementia and (3) the perception of coping resources available if developing dementia (see figure 2). Our findings support this model as: (1) those with high dementia worry were more likely to identify with those with dementia; (2) those with high dementia worry viewed seriousness in relation to themselves, whereas those with low dementia worry viewed seriousness in relation to others; (3) those with low dementia worry had the perception of the presence of numerous relevant coping strategies, whereas those with high dementia worry did not. This original model has recently been supported by a qualitative study, in which they interviewed older adults and found themes supporting that perception of risk, seriousness and coping underlies dementia worry in this population.^45^ Kessler’s original model, however, does not give clear directions on how to positively alter individuals’ perceptions of these dimensions.

Importantly, our findings suggest that there also is a ‘self-other’ dimension, which is associated with the original dimensions (risk, seriousness and coping), thus providing new understanding of the reasons for dementia worry in older age. It appears to be the case that individuals with high dementia worry tended to relate issues of dementia to *the self*. Contrarily, those with low dementia worry tended to relate issues of dementia to *others*. We argue that this is associated with different interpretations of risk, seriousness and coping, as described in the previous paragraph, ultimately leading to different levels of dementia worry. The present study cannot provide evidence of the directional and causal relationship between the concepts, which should be investigated further. Nonetheless, our theory is supported by evidence suggesting a clear relationship between self-focused thinking and anxiety,^60 61^ and evidence showing that individuals with high dementia worry are likely to also have ageing anxiety,^62 63^ general anxiety^64^,^66^ and illness worry.^66^ More broadly, evidence supports the idea that individuals tend to organise their thinking in terms of self-other themes, which supports the reliability of this finding.^67 68^ People tend to view various concepts such as ‘having dementia’ or ‘having a mental health condition’ as something related to either themselves or to others, and depending on this thinking pattern, their views and behaviours around that concept change.^67 68^ As we will describe in more detail, we view this as a relevant target for interventions of dementia worry.

### Interventions to increase metacognitive accuracies and decrease dementia worry

As described throughout this paper, metacognitive beliefs and dementia worry are associated with many negative health outcomes including an impact on mental health and importantly an increased risk of dementia.^821 23 25 69^,^73^ Identifying effective interventions for these types of negative thinking patterns is therefore crucial both for the health of the individual and the burden of the healthcare system. First, we encourage the use of interventions focused on improving metacognitive abilities as previous research has found this to be successful in individuals with subjective memory complaints.^74^,^76^ We encourage this to be extended to individuals with high dementia worry. Second, while national interventions focusing on increasing the awareness and knowledge of dementia have been frequently used,^77^,^79^ our data might suggest these are not always effective for many individuals. Increased knowledge of dementia does not appear to be associated with decreased dementia worry.^80 81^ Instead, based on our findings, interventions focusing on a healthy perception of ‘normal ageing’ and suitable comparison techniques may be more beneficial in decreasing dementia worry and negative metacognitive beliefs. This is also supported by previous evidence showing that negative stereotypical beliefs of ageing have negative effects on both metacognitive beliefs and dementia worry.^42 65 82 83^

In addition, our findings support the importance of decreasing negative self-focused thinking. Here, we propose psychological interventions focused on decreasing self-focused thinking. For instance, previous research has found that Cognitive Behavioural Therapy (CBT) focusing specifically on reducing self-focused thinking leads to reduced general anxiety,^84 85^ and some preliminary studies even suggest that general CBT can help decrease worries of dementia among older adults.^86^ Thus, combined with the findings of our study, it seems promising that CBT targeting self-focused thinking may be particularly effective in reducing dementia worry in older age. Similarly, mindfulness^87 88^ and meditation[Bibr R89]-focused^89^ interventions, which also aim to change self-referential thinking patterns,^90^,^92^ may reduce dementia worry. In the current study, we did not measure mental health or mood, but it is likely that those with high dementia worry and negative metacognitive beliefs generally have a tendency for negative thinking patterns, anxiety and depression. Establishing the relation and causal relationship between mental health issues and negative thinking patterns will be an important step in identifying the specific targets of these interventions.

These types of interventions could be offered to individuals with a close relationship to individuals with dementia, because these are at a particular risk of high dementia worry. In our research, we found that those with high dementia worry more often told personal stories of individuals close to them with dementia. This is supported by previous research showing that exposure to dementia in the family is related to a higher risk of dementia worry and subjective cognitive decline,^6 41 44 66 93 94^ and may be of particular relevance if the relatives know of a genetic disposition to dementia.^6 42 94^ One potential obstacle to identifying these individuals has been described by Werner *et al*,^7^ who proposed to expand the original model by Kessler and Bowen.^44^ They argued that some individuals will avoid consulting professionals or thinking about dementia, due to their worries^95 96^ (see figure 2). In our study, over half of those with high dementia worry had discussed their worries with a health professional. This is in line with an argument proposed by Edmonds *et al*,^97^ describing how some individuals may maladaptively seek help, which can lead to misdiagnoses. We would propose that there might be two groups of individuals with high dementia worry; those who ‘avoid’ and those who ‘seek’ help (see figure 2). We argue that this distinction is important to keep in mind as both groups have separable and opposing risks.

### Limitations

This study used a qualitative framework to investigate metacognitive beliefs between individuals with different levels of dementia worry. We acknowledge the limitations of the current study. First, participants were aware of whether they were in a ‘low dementia worry’ or a ‘high dementia worry’ group which may have influenced the tones and narratives in the group discussion and may have caused greater differences between the groups. This was done to assure people that they were discussing in a group of people who had a similar experience of worry as themselves, and so the aim of disclosing this was to maximise comfortable discussion. Second, the current analysis sheds light on the potential presence of two behavioural groups: those who seek and those who avoid dementia-related situations. In the case of the presence of two groups, the findings of our study are limited to those who ‘seek’, as these were individuals who had volunteered to be part of a focus group discussion. Individuals who ‘avoid’ would be assumed to avoid a study like this. Furthermore, as mentioned, one individual scored below the threshold of MCI on the Tele-MACE. The quantitative statistics were re-calculated without this individual, which did not change the pattern of results. However, this may have influenced the discussion in the respective focus group. In addition, it should be noted that some participants, particularly in the high dementia worry group, may be defined as having subjective cognitive decline. For example, those who have discussed memory issues with their family or medical professionals (n=8) would, in some research, be described as having subjective cognitive decline as they have these beliefs in the absence of a memory impairment on the Tele-MACE.^10 11^ An increasing amount of research suggests that such individuals may have subtle cognitive decline, undetectable on tests such as the Tele-MACE.^98^,^100^ Thus, some individuals in the high dementia worry group may in fact experience more memory slips than those with low dementia worry, despite this being undetectable to the measure employed here. This difference may have directly influenced the discussion and increased the difference between the two groups.

Turning to the quantitative measures in this study, common mental health traits such as anxiety, depression and family history of dementia, which have been shown to be highly correlated with dementia worry.^7 39^ Finally, the sample was underpowered for the quantitative analysis presented in this study, and so this should be interpreted cautiously. The primary goal of this study was the qualitative analysis of focus group discussions.

## Conclusion

In conclusion, our findings suggest that older individuals appear to employ comparative metacognitive strategies in order to build their metacognitive beliefs, and we propose that these strategies are a useful target for interventions. Specifically, we encourage knowledge-based interventions that encourage positive and effective social comparisons rather than focus on providing information on dementia occurrence. In addition, there appears to be a self-other dimension influencing the previously proposed dimensions of dementia worry, with a strong self-focus being associated with increased worry. Targeting this focus is likely to be useful in ameliorating harmful levels of dementia worry and could be supported through adapting current therapeutic tools for anxiety disorders.^84 85^ We argue that these should be seen as part of dementia prevention methods and could be offered to relatives and close associates of individuals with dementia.

## Supplementary material

10.1136/bmjopen-2024-097002 online supplemental file 1

## Data Availability

No data are available.
