# Supplementary material for: How do metacognitive beliefs about memory differ between older adults with low and high dementia worry? A focus group study
Source: BMJ Open. 2025 Oct 2;15(10):e097002. doi: 10.1136/bmjopen-2024-097002 (PMC12496098; doi:10.1136/bmjopen-2024-097002)
Supplement: online supplemental file 1 [file bmjopen-15-10-s001.docx]

**Supplementary Material**

**Supplementary Material 1:** Full script and protocol for focus group discussion.

**Introduction:**

Hello all, Welcome.

Thank you all for participating in the initial phone call and joining us here for the focus group discussion. We are researching Memory beliefs with King’s College London.

Researchers will introduce themselves.

We will briefly go through some general regulations for today.

Participants will be encouraged to wear masks and to social distance. They will be told that the room has been well ventilated and cleaned before the meeting and that everyone in the room is fully vaccinated. Participants will be told that the groups were arranged based on how worried participants were about their own memory in our questions on the telephone. This will be made explicit so that people are comfortable with expressing their opinions in the group. Then, the participants will be reminded that the focus group meetings are confidential, and they should not share information about the other participants or what was discussed at the meeting. Participants are asked to follow King’s College London Safe space policies, meaning that they should be respectful to each other. They are also reminded that they do not need to share personal information about themselves or anything that makes them feel uncomfortable. It will be outlined that they will discuss the experiences of fictional characters. The researchers will also acknowledge that the topic can be distressing for some, highlighting the importance of being mindful of others, and that if someone needs a break, they can let the researchers know, or silently leavewithout giving reason. Lastly, the researchers will describe how the focus group will proceed: First an icebreaker, then the vignette, which will guide the discussion, and in the end a few questions to wrap up. This will take approximately 1 hour. It will also be highlighted that the aim of the research is to learn from the participants, and we therefore hope they will engage actively, and that there are no right or wrong answers.

**Icebreaker:**

Researcher introduces themselves first by answering the ice-breaker question.

Participants introduce themselves to each other.

Icebreaker question:

1.Which animal best describes your morning?

OR

2.Who is your favourite musician?

“We will now go on to the main part of this focus group discussion. I will read out a story of a fictional character named Mary. This is also the story you have on the paper you just received. Please listen to the story, and afterwards I will ask you to discuss different aspects of the story. Please remember that there are no right or wrong answers, and you are not required to share information about your own life. However, if you want to you are welcome to do so, but please do be mindful of the sensitive nature of the topic and not to share any distressing or traumatic events as this might be uncomfortable both for you and the others in the room.”

(All participants will each receive a printed-out version of the vignette).

**Vignette:**

Mary lives in London, and she has two grandchildren whom she loves dearly. She visits them each Saturday, and they usually go to the park to look at the ducks. Mary has to take two tubes to get to their place, but she knows the route and is never late. She always makes sure to bring a few sweets that she gives to the grandchildren when they are in the park, so their mother doesn’t notice. This Saturday her tube journey goes smoothly, and her grandchildren are putting on their jackets when she arrives. On their way to the park, they meet an old friend of Mary’s, Paul, who is walking his dog. Mary and Paul talk for a bit, while the grandchildren pet the dog. Afterwards, they find the pond, which is crowded with ducks. The grandchildren ask Mary what the baby ducks are called, and she says they are called ‘ducklings’, and they count that there are seven of them. After a while Mary puts her hands in her pockets to get the sweets for her grandchildren but realizes that she has forgotten them. The grandchildren laugh, and say it is okay. To make up for the missing sweets, Mary suggests that they buy ice cream instead, before they go home.

I will give you 5 minutes to re-read the vignette before we proceed to the discussion. Don’t worry this is not a memory assessment, we will just have an overall discussion about your interpretation of the vignette.

**Follow-up Questions:**

1. How do you think Mary feels about forgetting the sweets?

2. Do you think forgetting the sweets will affect Mary in any way next time she takes her grandchildren to the park?

3. Do you think forgetting the sweets affects how Mary thinks about her ability to remember?

4. Do you think Mary would feel differently had she forgotten the name of her old friend Paul?

5. Do you think Mary would have felt differently had she forgotten her tube route?

6. Do you think if Mary had forgotten that baby ducks are called ducklings she would have been more of less concerned?

7. How old do you think Mary is? How does this influence your answers?

8. Do you think you would have answered differently if Mary had forgotten something in a work context instead of near her grandchildren? If for instance she had forgotten the keys to the charity shop where she volunteers.

9. If Mary starts worrying about her memory, do you think there is a way for her to re-gain her confidence?

Now we have completed the questions relating primarily to Mary and now we will move on to some general questions about memory.

**Other potential questions, if the discussion about the vignette runs short:**

1. Some individuals think they are bad at remembering even though they are not. Why do you think that is?

2. Based on our discussions today, can you list the two most important types of events that you think would cause someone to doubt their own memory ability?

3. Based on our discussions today, which aspect of a person’s life do you think is most affected by doubting their own memory?

You have all brought up great points so thank you very much. The focus group is now coming to an end so we will wrap up with two final questions.

**Final Question:**

1. What do you think was the most important or interesting point that was brought up today?

2. Are there any final remarks? Something that you want to add?

**Wrap-up:**

The researchers will thank the participants for their time and give them their reimbursements. The researchers will also repeat what will happen next with the data: That it will be transcribed, analysed, and then included in a BSc and PhD thesis, and potentially written publications. For those of you who requested a write up of the results, they will be sent to you upon completion. If you change your minds, a follow-up email, will instruct you on how to contact the research team about this. The participants will also be told that this follow-up email will contain a debrief of the study, and that if the discussion has been distressing to any of them, they can contact their GP or the researchers for further signposting. Participants will also be reminded that the contact details of the research team are on the information sheet they received prior to the focus group discussion and will also be in the follow-up email.

**Supplementary Material 2**: The Consolidated Criteria for Reporting Qualitative Research (COREQ) checklist

| Domain 1 | Research Team and reflexivity |  |
| --- | --- | --- |
| Personal Characteristics |  |  |
| Interviewer/facilitator | Which author/s conducted the interview or focus group? | Primarily the first Author AEL, with help from undergraduate students. |
| Credentials | What were the researcher’s credentials? E.g. PhD, MD | PhD and BSc students |
| Occupation | What was their occupation at the time of the study? | Students |
| Gender | Was the researcher male or female? | All were female |
| Experience and training | What experience or training did the researcher have? | Psychology degree as well as specific training to qualitative research and focus group discussion, organised both by the university and the research team. |
| Relationship with Participants |  |  |
| Relationship established | Was a relationship established prior to study commencement? | No |
| Participant knowledge of the interviewer | What did the participants know about the researcher? e.g. personal goals, reasons for doing the research | The participant information sheet sent to participants prior to participation, informed of the general goal of investigating how older adults think about their memory abilities. In addition, they knew that they were divided into groups based on their level of dementia worry. |
| Interviewer characteristics | What characteristics were reported about the interviewer/facilitator? e.g. Bias, assumptions, reasons and interests in the research topic | It was mentioned that focus group discussions were used partly to decrease the influence of the researcher on the discussions. |
| Domain 2 | Study Design |  |
| Theoretical Framework |  |  |
| Methodological orientation and Theory | What methodological orientation was stated to underpin the study? e.g. grounded theory, discourse analysis, ethnography, phenomenology, content analysis | Inductive Thematic Analysis in accordance to (Braun & Clarke, 2006, 2012, 2022). |
| Participant selection |  |  |
| Sampling | How were participants selected? e.g. purposive, convenience, consecutive, snowball | Convenience sampling. |
| Method of approach | How were participants approached? e.g. face-to-face, telephone, mail, email | The study was advertised through a newsletters, participants contacted the researchers if they were interested. |
| Sample size | How many participants were in the study? | 35 participants. |
| Non-participation | How many people refused to participate or dropped out? Reasons? | None. |
| Setting |  |  |
| Setting of data collection | Where was the data collected? e.g. home, clinic, workplace | At King’s College London university. |
| Presence of non-participants | Was anyone else present besides the participants and researchers? | No. |
| Description of sample | What are the important characteristics of the sample? e.g. demographic data, date | The different levels of dementia worry. |
| Data Collection |  |  |
| Interview guide | Were questions, prompts, guides provided by the authors? Was it pilot tested? | All questions are provided in the method section. No pilot test was run. |
| Repeat interviews | Were repeat interviews carried out? If yes, how many? | No. |
| Audio/visual recording | Did the research use audio or visual recording to collect the data? | Audio recordings were made. |
| Field notes | Were field notes made during and/or after the interview or focus group? | Field notes were taken by the main researcher AEL after the focus group discussion and throughout data analysis. |
| Duration | What was the duration of the interviews or focus group? | 1 hour each. |
| Data saturation | Was data saturation discussed? | Yes |
| Transcripts returned | Were transcripts returned to participants for comment and/or correction? | No. |
| Domain 3. | Analysis and Findings. |  |
| Data analysis |  |  |
| Number of data coders | How many data coders coded the data? | One |
| Description of the coding tree | Did authors provide a description of the coding tree? | No, but a table with themes and sub-themes were provided. |
| Derivation of themes | Were themes identified in advance or derived from the data? | Derived from the data |
| Software | What software, if applicable, was used to manage the data? | NVivo was used. |
| Participant checking | Did participants provide feedback on the findings? | No. |
| Reporting |  |  |
| Quotations presented | Were participant quotations presented to illustrate the themes / findings? Was each quotation identified? e.g. participant number | Yes. |
| Data and findings consistent | Was there consistency between the data presented and the findings? | Yes. |
| Clarity of major themes | Were major themes clearly presented in the findings? | Yes. |
| Clarity of minor themes | Is there a description of diverse cases or discussion of minor themes? | Minor themes were discussed but single individuals were not pointed out as diverse cases. |

**Supplementary Material 3:** Quantitative measures of cognitive and metacognitive abilities and behaviours, excluding one participant who scored at a level of Mild Cognitive Decline on the Tele-MACE. As seen no difference is found from the test when including this individual.

| Tele-MACE (SD) | 23.27 (2.05) | 23.92(1.11) | 23.52(1.77) | .693 |
| --- | --- | --- | --- | --- |
| Dementia Worry (SD) | 18.14 (4.54) | 36.08(4.94) | 24.8 (9.93) | <.001* |
| Memory Confidence (SD) | 3.18 (0.73) | 3(1.08) | 3.11(0.87) | .606 |
| Discussion w. relatives (SD) | 1.95(0.84) | 2.77(1.09) | 2.26(1.01) | .025* |
| GP: Yes (No) | 0 (22) | 8 (5) | 8(27) | .010* |

*Mean and standard deviations of reports and performance on quantitative measures; dependent 2-group Wilcoxon Signed Rank test were performed for numeric variables (Age, Total Mace, Dementia Worry, Memory Confidence and Relative), Chi2 tests were performed for the remaining categorical variables.*
