# Supplementary material for: How do metacognitive beliefs about memory differ between older adults with low and high dementia worry? A focus group study
Source: BMJ Open. 2025 Oct 2;15(10):e097002. doi: 10.1136/bmjopen-2024-097002 (PMC12496098; doi:10.1136/bmjopen-2024-097002)
Supplement: Supplementary file 2 [file bmjopen-2024-097002.reviewer_comments.pdf]

PEER REVIEW HISTORY

BMJ Open publishes all reviews undertaken for accepted manuscripts. Reviewers are asked to complete a checklist review form and are provided with free text boxes to elaborate on their assessment. These free text comments are reproduced below.

ARTICLE DETAILS

Title (Provisional)

How do metacognitive beliefs about memory differ between older adults with low and high dementia worry? A focus group study

Authors

Lund, Astrid Emilie; Freiin von Stein zu Nord- und Ostheim, Alexandra; Ridley, Hayley; Bobyreva, Kamilla; Foster, Juliet L. H.; Russell, Charlotte

---

VERSION 1 - REVIEW

**Reviewer**	**1**
**Name**	**Turner, Rachael**
**Affiliation**	**University of Nebraska System, Psychology**
**Date**	**30-Apr-2025**
**COI**	**None**

General Notes

1. Line numbers do not match the text

2. Contradictory page numbers (top left vs bottom right)

3. Inconsistency in formatting. Not all paragraphs are indented. Some numerical data is presented with '=' and some not. Spacing surround the '=' is inconsistent.

Introduction

4. Missing indention on paragraph two (pg. 5, line 37)

5. Normal and abnormal – consider changing to pathological, typical age-related decline (pg. 6, line 28)

6. You note that declines in episodic memory would be more likely to change one's metacognitive beliefs based on the fact that episodic memory decline is one of the first signs of AD. However, that would require the individual knows that is the first sign, which may not be true. You just discussed that different patterns of decline may cause difficulty in making metacognitive beliefs and you're assuming individuals know the first signs of AD is episodic memory decline. (pg. 6, line 28 – 42)

7. General metacognitive beliefs about one's general traits (pg. 6, line 47 – 48)

8. Describe what a 'maladaptive manner' would entail (pg. 6, line 52)

9. Misspelling – centering (pg. 6, line 56)

10. Is ‘inform’ the best descriptor (pg. 6, line 58). It seems strange as the metacognitive beliefs are their own beliefs, so you’re saying one is informing their own beliefs.

Method

11. Missing indention on both paragraphs of the qualitative methodology section (pg. 12, line 28 and 49)

Results

12. Determine that the sample is underpowered, but you don’t explain analyses you did to determine that (pg. 13, line 22).

Discussion

13. Please discuss why there appears to be a mismatch between the findings of the qualitative study and the results of the subjective beliefs about memory ability questionnaire.

14. You propose that self-other determines the view on seriousness, risk, and coping. However, this cannot be concluded based on the methodology used, as it is equally possible that these original dimensions are leading to either high or low dementia worry, and consequently influencing the focus on the self or others.

15. Discuss limitation of ‘diagnosis’ vs possible undiagnosed condition especially in relation to the participant that scored low enough on the Tele-MACE to indicate possible MCI.

16. Your acknowledgement of limitations is generally very brief. You previously mention that you are underpowered, yet this is not addressed in the limitations section. Additionally, the supplemental materials indicate that participants knew they were separated based on dementia worry levels. This could have influenced their perception of the questions as well as their conversations during the focus group.

**Reviewer**	**2**
**Name**	**Aihara, Yoko**
**Affiliation**	**Okayama University**
**Date**	**05-Jul-2025**
**COI**	**None**

Overall, I felt difficult to understand, perhaps due to the way it was written.

1. Abstract: In results part, no clearly understandable. The authors indicated that two themes were revealed but not indicated how these differ by the levels of worriness.

2. Introduction: P8, 40- The current study

This statement was duplicated in the method section.

2. Methods: 1) Participants: how and at where did the researchers recruite the participants?

2) Please review the structure of the method section. The procedure of the study should be written in the first part.

3) Were participants randomly divided into each groups? And please indicate the number of participants in each group (4-5 participants in each?)

4) p14, in the results section, statistical analytical methods should be written in methods section.

VERSION 1 - AUTHOR RESPONSE

Reviewer 1:
Dr. Rachael Turner, University of Nebraska System

Thank you for your thorough and helpful feedback. We have tried to incorporate all of your suggestions, as seen below.

General Notes

1. Line numbers do not match the text

Thank you for pointing this out. The line numbers that did not match are created by the submission portal, we cannot directly edit this. We apologize for the inconvenience. For this reason, we will not refer to line numbers in the text below, but hopefully the changes will be clear in the tracked-changes format.

2. Contradictory page numbers (top left vs bottom right).

Thank you, we believe this to be the same issue as above. We have made sure the numbers on our manuscript, and referred to in the text, are now on the bottom right – these will be the same as those referred to in the text so please follow those when reading. Sorry again for the inconvenience.

3. Inconsistency in formatting. Not all paragraphs are indented. Some numerical data is presented with '=' and some not. Spacing surround the '=' is inconsistent.

Thank you for pointing this out and please excuse these formatting errors. We have gone through the manuscript again to ensure consistency. Please see for instance pages 7, 8 and 13 for these changes.

4. Missing indention on paragraph two (pg. 5, line 37)
Thank you, this is added, along with other missing indention in other parts of the manuscript.

5. Normal and abnormal – consider changing to pathological, typical age-related decline (pg. 6, line 28)

Thank you for highlighting this point, we agree that the wording suggested is more appropriate and the text has been changed as follows on page 6:

One such reason for the high level of worry may be the difficulty in distinguishing typical age-related decline from pathological memory changes.

6. You note that declines in episodic memory would be more likely to change one's metacognitive

beliefs based on the fact that episodic memory decline is one of the first signs of AD. However, that would require the individual knows that is the first sign, which may not be true. You just discussed that different patterns of decline may cause difficulty in making metacognitive beliefs and you're assuming individuals know the first signs of AD is episodic memory decline. (pg. 6, line 28 – 42)

Thank you for pointing this out, this is a very useful point to clarify. To address your queries, we acknowledge that we do not expect the general public to know that what is termed 'episodic memory' in research appears to be the among the first cognitive system impacted by AD. However, there is evidence that older adults are aware of many symptoms of Alzheimer's Disease. We have added this clarification to the text - these changes can be found on page 6.

Knowledge of dementia varies between individuals and cultures, and greater awareness is encouraged [1, 2]. However, research suggests that many older adults know that incidents of forgetting what has happened or where one is, may be signs of dementia [3, 4]. Both of these are examples of episodic memory slips. Thus, it seems plausible that older adults may be particularly wary about changes of this type. Furthermore, if a person notices such changes, it is likely that they change their metacognitive beliefs about their ability to remember and may start to worry about dementia.

7. General metacognitive beliefs about one's general traits (pg. 6, line 47 – 48)

Thank you again, we see that this phrasing is unhelpful. We have changed the sentence on page 6 as follows:

When it comes to metacognitive beliefs about one's general traits, research suggests that one such metacognitive strategy is social comparison.

8. Describe what a 'maladaptive manner' would entail (pg. 6, line 52)

Thank you for helping make the manuscript more readable and accessible, we have added the following description (in red) on page 6:

If these comparisons are made in a maladaptive manner, such as overestimating the discrepancy between oneself and one's peers, it may lead to metacognitive inaccuracies.

9. Misspelling – centering (pg. 6, line 56)

The word has been changed to the US spelling "centering".

10. Is 'inform' the best descriptor (pg. 6, line 58). It seems strange as the metacognitive beliefs are their own beliefs, so you're saying one is informing their own beliefs.

We apologise, as this seem to be a spelling mistake. That is, we meant 'form' rather than 'inform'. The sentence has been changed as seen below (pg. 6), and hope it is understandable.

In particular, we were interested in investigating how older adults may **form** their metacognitive beliefs based on different types of memory information, such as information from experiences of episodic and semantic memory slips.

11. Missing indention on both paragraphs of the qualitative methodology section (pg. 12, line 28 and 49)

These indentations have been added.

12.(You) Determine that the sample is underpowered, but you don't explain analyses you did to determine that (pg. 13, line 22).

Thank you for highlighting this. We have re-done the power analysis and added the following text to the quantitative methodology section on page 12 to incorporate this detail:

Assuming a medium effect size of d=.05, the power to detect this difference in a two-tailed t-test was β=.28, well below the standard of β=.80. Conservative non-parametric tests (Wilcoxon Signed rank test and χ^2 tests) were employed to minimize these issues. The tests were used to ensure that the groups were appropriately matched and investigate differences between those with low and high dementia worry. Given the lack of power these results should be interpreted cautiously.

In addition, the following sentence was added to the qualitative methodology section (pg. 12) to highlight that the sample was not underpowered for the qualitative analysis.

The sample was appropriately powered to achieve thematic saturation [5, 6].

13. Please discuss why there appears to be a mismatch between the findings of the qualitative study and the results of the subjective beliefs about memory ability questionnaire.

Thank you, this is a very useful point. A key issue here is that the focus group analysis allows us to probe differences and distinctions that are not apparent in a single question. We think this is an important strength of the qualitative design here and have added the following to the Discussion on page 21 to 22:

Interestingly, the 1-item questionnaire on subjective beliefs about memory ability, found no statistically significant differences between the Low and High dementia worry participants. This question asked participants to rate their ability to remember. The fact that there is evidence of lower confidence around memory abilities among those with high worry in the qualitative analysis but not the quantitative analysis may result from a number of reasons. As previously mentioned, the sample was not powered for quantitative analyses but rather qualitative analysis. Issues around power for quantitative analysis are also exacerbated by the use of a 1-item questionnaire as there are a small amount of data points. Conversely, the qualitative data is richer, allowing us to explore the nuanced differences between the two groups. It is worth noting that this limitation is common in this field of research; many studies investigating insight into memory abilities in older adults rely on 1-item questionnaires similar to the one used in this study [7-9]. The current study highlights the need for richer research to assess subjective memory beliefs.

14. You propose that self-other determines the view on seriousness, risk, and coping. However, this cannot be concluded based on the methodology used, as it is equally possible that these original dimensions are leading to either high or low dementia worry, and consequently influencing the focus on the self or others.

Thank you for pointing this out, it of course correct that we cannot establish causation and we apologise for this mistake. We have changed Figure 2 and the text as detailed below on page 22-24:

In the Discussion the following changes has been made to the section now called "*A self-other dimension as an important mechanism of Dementia Worry*".

Several small changes were made to the section to change the language from causal to associative such as "mechanism" or "associated with" rather than "causes" and "leading to" throughout these pages. In addition, the following was added:

Importantly, our findings suggest that there also is a 'self-other' dimension, which is associated with

the original dimensions (risk, seriousness and coping),

[..]

The present study cannot provide evidence of the directional and causal relationship between the concepts, which should be investigated further.

[…]

More broadly, evidence supports the idea that individuals tend to organise their thinking in terms of self-other themes, which supports the reliability of this finding [10, 11]. People tend to view various concepts such as "having dementia" or "having a mental health condition" as something related to either themselves or to others, and depending on this thinking pattern their views and behaviours around that concept changes [10, 11].

In the conclusion on page 26, we have also changed the following:

In addition, there appears to be a self-other dimension **influencing** the previously proposed dimensions of dementia worry, with a strong self-focus being associated with increased worry.

The figure has also been updated to illustrate that no causal relationship has been established and that the relationships may be bidirectional. To do so all arrows are bi-directional, and an extra arrow has been added between the Self-Other component and Dementia worry.

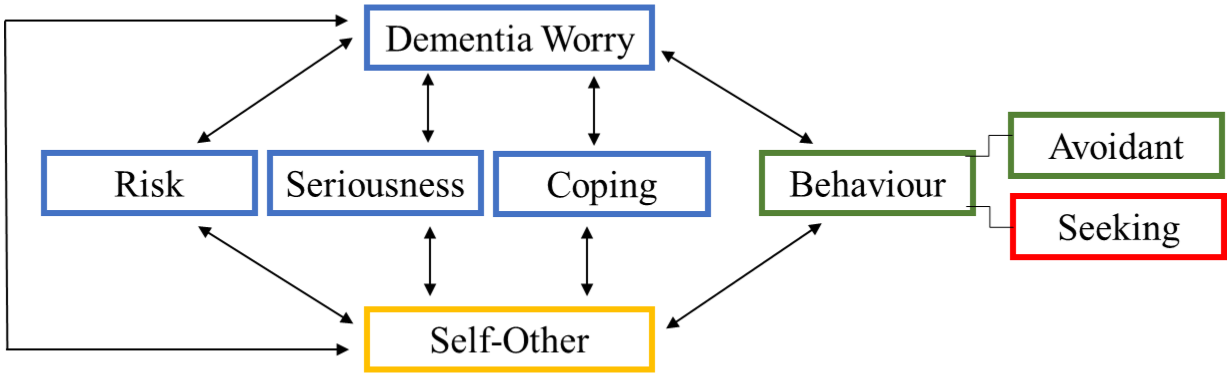

15. **Discuss limitation of 'diagnosis' vs possible undiagnosed condition especially in relation to the participant that scored low enough on the Tele-MACE to indicate possible MCI.**

Thank you for pointing out this important factor. The following has been added to the limitations section on page 25:

Furthermore, as mentioned, one individual scored below the threshold of MCI on the TELE- MACE. The quantitative statistics were re-calculated without this individual which did not change the pattern of results. However, this may have influenced the discussion in the respective focus group. In addition, it should be noted that some participants, particularly in the high dementia worry group may be defined as having subjective cognitive decline. For example, those who have discussed memory issues with their family or medical professionals (N=8) would, in some research, be described as having subjective cognitive decline as they have these beliefs in the absence of a memory impairment on the TELE-MACE [12, 13]. An increasing amount of research suggests that such individuals may have subtle cognitive decline, undetectable on tests such as the TELE-MACE [14-16]. Thus, some individuals in the high dementia worry group may in fact experience more memory slips than those with low dementia worry, despite this being undetectable to the measure employed here. This difference may have directly influenced the discussion and increased the difference between the two groups.

16. Your acknowledgement of limitations is generally very brief. You previously mention that you are underpowered, yet this is not addressed in the limitations section. Additionally, the supplemental materials indicate that participants knew they were separated based on dementia worry levels. This could have influenced their perception of the questions as well as their conversations during the focus group.

Thank you for further highlighting limitations and increasing the transparency of this manuscript. We have added the following sections to the limitation section of the discussion, on page 25-26:

Firstly, participants were aware of whether they were in a "low dementia worry" or a "high dementia worry" group which may have influenced the tones and narratives in the group discussion and may have caused greater differences between the groups. This was done to assure people that they were discussing in a group of people who had a similar experience of worry as themselves, and so the aim of disclosing this was to maximise comfortable discussion.

[…]

Turning to the quantitative measures in this study, common mental health traits …Finally, the sample was underpowered for the quantitative analysis presented in this study, and so this should be interpreted cautiously. The primary goal of this study was the qualitative analysis of focus group discussions.

Reviewer: 2
Dr. Yoko Aihara, Okayama University

Thank you for taking the time to read the paper and provide helpful feedback. We have tried to incorporate all your suggestions into our edits, as detailed below. As you will see, your article "Dementia literacy and willingness to dementia screening" also helped us answer a piece of feedback given by Reviewer 1, thank you for this work.

To clarify, as mentioned above, we are referring to the page numbers in the bottom-right corner.

Comments to the Author:
Overall, I felt difficult to understand, perhaps due to the way it was written.

Thank you for pointing this out, it is a key priority for us that the message of this paper is accessible and comes across clearly to the reader. We apologise that this may not have been the case in all parts of the manuscript. We also acknowledge that it may be difficult for us to evaluate the readability of our own written text. Therefore, to address this issue we asked two researchers who were not involved in this study to read through the manuscript and highlight to us parts that they found more challenging to understand. One reader was not a native English speaker, and one was. Based on their feedback we examined which parts of the manuscript could be edited to increase readability. We hope this helps. If there are further specific parts that you still find may be difficult to understand, please let us know where and we will re-word.

The following changes were made to the manuscript based on the feedback from the two readers. We hope that this, along with the other changes, have improved the clarity of the manuscript.

We made the following changes to the Qualitative Methodology section on page 12: Codes

were created separately for each group (Low worry and High worry).

[…]

Generating themes: Initial subthemes were created first for each group (Low and High worry) and then overarching themes were created covering both groups.

Furthermore, numbers were added Table 3 on page 14 to match the numbering used in the Results

section below.

Table 3. Themes and sub-themes		
**Themes**	**Group**	**Sub-themes**
1. The question of normality	Shared	1.1 Strategies for defining normality<br>1.2 Characteristics and threshold of normality<br>1.3 Preservation of normality
	Low Worry	1.4.a The normalisation of forgetting
	High Worry	1.4.b The normalisation of fear
2. The importance of the self or others	Low Worry	2.1.a An issue relevant to others<br>2.2.a Consequences of Dementia on others<br>2.3.a Coping with potential Dementia
	High Worry	2.1.b An issue relevant to oneself<br>2.2.b Consequences of Dementia on the self<br>2.3.b Fear of potential Dementia

1. Abstract: In results part, no clearly understandable. The authors indicated that two themes were revealed but not indicated how these differ by the levels of worriness.

Thank you for highlighting this. The Abstract is the first impression of the manuscript, and we appreciate your feedback as we hope to clearly convey our findings. We have changed the result section, as seen below:

Results: Thematic Analysis revealed two key themes. First, older adults appear to base their definition of 'normality' of their own memory on *comparisons*. These comparisons were between themselves and others; and between themselves now and their own past self. Despite similar strategies to define 'normality', those with high dementia worry had stricter definitions of what they determined as normal. The second theme described narratives around the "self" and the "other". There was a difference between those with high versus low worry; those with high worry had a strong focus on the "self", while those with low dementia worry focused on "others".

2. Introduction: P8, 40- The current study
This statement was duplicated in the method section.

Thank you for highlighting this apparent duplication. Indeed, we understand it can be frustrating to read repetitive text. We have had a look again at these parts of the manuscript. We do wish to maintain a short description of the study design in the Introduction as this is relatively common practice and provides the reader with an overview before reading the details in the Method. To edit we have removed this as a separate sub-heading, and the section has been shortened to make it less repetitive of the later sections. The following edits can be seen on page 7.

Here, we conducted focus groups with adults aged 65 and above. In order to understand how different levels of worry might lead to differing perceptions of memory, separate focus groups were carried out with individuals who had respectively low and high worry levels around dementia. All focus groups were structured around a fictional vignette. The discussions were analysed with Thematic Analysis [17, 18], as this is a widely used analysis framework in qualitative work, and allows for the flexibility and depth of analysis we aimed to achieve.

2. Methods:

1) Participants: how and at where did the researchers recruit the participants?

Thank you for highlighting this issue, we have added the following information on page 7:

Participants were recruited through internal advertisement at King's College London and external advertisement at through University of the Third Age, UK.

2) Please review the structure of the method section. The procedure of the study should be written in the first part.

Thank you for your feedback, we are hesitant to move the Procedure to the beginning of the Method section. In re-considering the structure, we see that moving the procedure would mean that readers do not know what, for instance, the TELE-MACE refers to and so it might cause greater confusion. As it is not uncommon practice to structure a Method section in this way. Furthermore, we do not see any requirements from the journal nor from the editor who read this paper. We hope this makes sense alongside the edits made above. However, if this remains an issue, we will of course change it.

3) Were participants randomly divided into each groups? And please indicate the number of participants in each group (4-5 participants in each?)

Thank you again for helping us improve the manuscript by pinpointing missing points in our description of the methods. Once we knew which broad category of focus group (i.e. High or Low Worry) a participant would be applicable to take part in, we gave them a series of potential times and dates to facilitate organising the sessions and so participation in a specific group on a particular day was based on their own availability. We have added the following information to the participant section on page 7-8:

Twenty-two participants were identified as having Low worry (mean=18.14, SD=4.54) and were divided into another four focus groups (4-7 participants in each group). Thirteen participants were identified as having High Worry as they had scores over 30 (out of 60) (mean=36.08, SD=4.94) and were divided into three focus groups (3-4 participants in each group). Participants were added to a group based on dementia worry level and availability for partaking in focus group discussions.

4) p14, in the results section, statistical analytical methods should be written in methods section.

Thank you for your feedback. The following has been added to the Methods section (pg. 12) and removed from the Results section.

Quantitative Methodology:

Quantitative differences between the two groups for the questionnaires and cognitive assessment were analysed. It is important to note that the aim of the current study was to examine qualitative differences between these groups, the quantitative results should be considered in this light, as the sample is slightly skewed (more people with Low Worry) and underpowered for quantitative analyses. Assuming a medium effect size of d=.05, the power to detect this difference in a two-tailed t-test was β=.28, well below the standard of β=.80. Conservative non-parametric tests (Wilcoxon Signed rank test and χ^2 tests) were employed to minimize these issues. The tests were used to ensures that groups were appropriately matched and investigate differences between those with low and high dementia worry, while this should be interpreted cautiously.

1. Aihara, Y. and K. Maeda, *Dementia literacy and willingness to dementia screening.* International journal of environmental research and public health, 2020. **17**(21): p. 8134.
2. Livingston, G., et al., *Dementia prevention, intervention, and care: 2024 report of the Lancet standing Commission.* The Lancet, 2024. **404**(10452): p. 572-628.
3. Jorge, C., et al., *Level of understanding of Alzheimer disease among caregivers and the general population.* Neurología (English Edition), 2021. **36**(6): p. 426-432.

4. McParland, P., et al., *Dementia knowledge and attitudes of the general public in Northern Ireland: an analysis of national survey data.* International Psychogeriatrics, 2012. **24**(10): p. 1600-1613.
5. Guest, G., A. Bunce, and L. Johnson, *How many interviews are enough? An experiment with data saturation and variability.* Field methods, 2006. **18**(1): p. 59-82.
6. Namey, E., et al., *Evaluating bang for the buck: a cost-effectiveness comparison between individual interviews and focus groups based on thematic saturation levels.* American Journal of Evaluation, 2016. **37**(3): p. 425-440.
7. Pike, K.E., et al., *Subjective cognitive decline: level of risk for future dementia and mild cognitive impairment, a meta-analysis of longitudinal studies.* Neuropsychology review, 2022. **32**(4): p. 703-735.
8. Wolfsgruber, S., et al., *Cerebrospinal fluid biomarkers and clinical progression in patients with subjective cognitive decline and mild cognitive impairment.* Journal of Alzheimer's Disease, 2017. **58**(3): p. 939-950.
9. Cova, I., et al., *Self-awareness for memory impairment in amnestic mild cognitive impairment: A longitudinal study.* American Journal of Alzheimer's Disease & Other Dementias®, 2017. **32**(7): p. 401-407.
10. Marková, I., *Dialogicality and social representations: The dynamics of mind*. 2003: Cambridge University Press.
11. Walsh, D. and J. Foster, *A contagious other? Exploring the public's appraisals of contact with 'mental illness'.* International journal of environmental research and public health, 2020. **17**(6): p. 2005.
12. Jessen, F., et al., *A conceptual framework for research on subjective cognitive decline in preclinical Alzheimer's disease.* Alzheimer's & Dementia, 2014. **10**(6): p. 844-852.
13. Jessen, F., et al., *The characterisation of subjective cognitive decline.* The Lancet Neurology, 2020. **19**(3): p. 271-278.
14. Buckley, R.F., et al., *Subjective cognitive decline from a phenomenological perspective: a review of the qualitative literature.* Journal of Alzheimer's Disease, 2015. **48**(s1): p. S125- S140.
15. Wulff, H., *The two cultures of medicine: objective facts versus subjectivity and values.* Journal of the Royal Society of Medicine, 1999. **92**(11): p. 549-552.
16. Leder, D., *Clinical interpretation: the hermeneutics of medicine.* Theoretical medicine, 1990. **11**: p. 9-24.
17. Braun, V. and V. Clarke, *Thematic analysis: A practical guide*. 2022, London, England: Sage Publications Sage UK.
18. Braun, V. and V. Clarke, *Using thematic analysis in psychology.* Qualitative research in psychology, 2006. **3**(2): p. 77.
